# Sustainability of organic zucchini in Mediterranean environment: an on‐farm experimentation

**DOI:** 10.1002/jsfa.14357

**Published:** 2025-05-12

**Authors:** Gaetano Roberto Pesce, Salvatore Alfio Salicola, Claudia Formenti, Gaetano Pandino, Giovanni Mauromicale, Sara Lombardo

**Affiliations:** ^1^ Department of Agronomy, Food, Natural Resources, Animals and Environment – DAFNAE University of Padua, Agripolis Campus Legnaro Italy; ^2^ Department of Agriculture, Food and Environment – Di3A Università degli Studi di Catania Catania Italy

**Keywords:** *Cucurbita pepo*, fertilization management, greenhouse tunnels, irrigation management, Mediterranean climate, organic farming, participatory approach

## Abstract

**BACKGROUND:**

Excess inputs are commonly applied to high‐value crops to ensure high performance. This study hypothesizes that farmers can reduce inputs without compromising yields and aims to investigate the effects of varying irrigation and fertilization strategies on two zucchini genotypes (‘Logos’ and ‘Atlantis’) organically grown under greenhouse tunnels in southern Italy over two seasons. Conducted on a large scale within an on‐farm experimentation framework, this research compared two different irrigation volumes (the farmer's experience‐based volume *vs.* a 25% reduction) and two nitrogen fertilization rates (the farmer's usual rate *vs.* a ~50% reduction).

**RESULTS:**

An average reduction of 550 m^3^ ha^−1^ of irrigation water led to a yield decrease of 3.0% (57.4 *vs.* 55.7 t ha^−1^), while a reduction in nitrogen fertilization (−156 kg ha^−1^ of N) resulted in a yield decrease of 3.3% (57.5 *vs.* 55.6 t ha^−1^). In light of these modest yield reductions, significant increases in irrigation water productivity (+33%) and fruit nitrogen use efficiency (+75%) were observed. The physical and color characteristics, along with the mineral composition of the fruits, were primarily influenced by the growing season and, to a lesser extent, by the genotype, while inputs had little to no effect.

**CONCLUSION:**

This paper offers insights into sustainable zucchini production, demonstrating that resource‐efficient farming can respond to environmental and economic challenges while maintaining satisfactory yields and fruit quality. The study highlights the effectiveness of a participatory approach as a means to generate reliable results for researchers while also providing outcomes that are directly applicable to farmers. © 2025 The Author(s). *Journal of the Science of Food and Agriculture* published by John Wiley & Sons Ltd on behalf of Society of Chemical Industry.

## INTRODUCTION

The Zucchini group belongs to the summer squash species (*Cucurbita pepo* L. subsp. *pepo*) and descends from one of the three major plants, together with maize and common bean, that were first domesticated in Mesoamerica 10 000 years ago.^1^ The *Cucurbita* genus was introduced into Italy from the New World by the mid‐16th century, but zucchini was bred near Milan, possibly around 1850.^2^ *Cucurbita pepo* is a vascular plant with flowers and seeds (Angiospermae); the dominant colors of the perianth are yellow or orange. The fruit is a pepo, with variable shape, size, consistency and external color (generally shades of green). The cultivation of zucchini in unheated greenhouses has become widespread in Italy and, in particular, in Sicily (southern Italy), because the mild winters typical of the Mediterranean climate allow out‐of‐season production with low energy inputs.^3^

Other factors have contributed to the increase of zucchini production in Sicily in recent years, such as modernization of agricultural practices and technologies, the introduction of new varieties that are more resistant to adversities, and better alignment with market demand. In addition, Sicilian zucchinis are not only demanded for domestic consumption, but are also exported across Europe and beyond.^4^ Organic agriculture aligns with the European Green Deal's objectives for sustainable food systems by 2030. Recent EU regulations bolster organic farming through financial incentives, leading to an increase in the number of organic producers.^5^ In 2022, Europe had 18.5 million hectares of organic farmland. In the EU, retail sales of organic products reached 45.1 billion euros, accounting for 34% of the global organic market.^5^ Per capita spending on organic products has doubled in Europe over the past decade, reflecting heightened consumer concern for health and environmental issues.^5^

Unfortunately, there are no specific official statistics on organic zucchinis in Italy, however it is worth pointing out that Italy is the third European country in terms of areas dedicated to organic farming (more than 2 million hectares in the 2019–2021 period)^6^ and, currently, approximately 25% of this area is in Sicily.^7^ This testifies to the importance of organic farming in the agri‐food economy of the island.

Even though yields do not increase linearly with increased nitrogen fertilization,^8^, ^9^, ^10^ the latter is often seen as an insurance against yield loss, because the vegetable production sector typically has a higher added value compared to other crops such as grasslands or arable crops.^11^ As a result, nitrogen fertilizer is frequently applied in excess of actual crop demand. In addition, irrigation water is an increasingly scarce resource, especially in arid and drought‐prone areas, such as those characterized by a Mediterranean climate.^12^, ^13^, ^14^ Agricultural experimentation typically relies on research conducted in small plots. This raises questions about the validity of inferences made from plot scale to farm scale, as crops at the field scale experience significant soil and topographic variability, which is often minimized at the plot scale. Additionally, there is potential bias introduced by using more favorable soils in plot experiments. The effects of scale discrepancy may be greater in organic systems due to their reduced effectiveness in controlling biotic stresses compared to conventional systems.^15^

Based on the aforementioned considerations, the hypothesis underlying the present work is that agronomic inputs for zucchini cultivation can be effectively reduced through careful management of water and nitrogen fertilization, without compromising yield or fruit quality. The aim of this work was to assess the yield, morphological parameters, and mineral profile responses of two commercial genotypes of zucchini, chosen for their suitability for winter cultivation in greenhouses and their long‐lasting shelf life, in an organic farm under tunnel greenhouses, subjected to two levels of nitrogen fertilization and two levels of irrigation over 2 years. The experiment took place on a large scale, in the real context of a farm, within the framework of a collaborative interaction between farmers and researchers. This study was farm‐centered, in other words originating from and responding to some needs of the farmer, who was looking for new practices to optimize fertilizers and irrigation water, for increasing efficiency and reducing environmental impact. All these elements combined make this work an on‐farm experimentation (OFE)^16^, ^17^ and contribute to filling the scientific gap regarding excessive inputs in organic horticulture, especially in empirical field‐scale studies. Soil water balances, growing degree days, irrigation water productivity and nitrogen use efficiency were also evaluated.

## MATERIALS AND METHODS

### Experimental site and climatic conditions

The experiment was conducted over a 2‐year period (2021 and 2023) in a farm located in the Ragusa province (36° 52′ 29.16″ N, 14° 31′ 13.72″  E, 105 m above sea level), where greenhouse zucchinis are traditionally grown. A physical–chemical analysis of the soil was carried out before planting the crop, in order to evaluate the fertilization and irrigation plans (Table 1). From the texture data the soil was classified as sandy loam, according to the US Department of Agriculture.^18^ The hydrological constants were calculated from the texture data, organic matter content and electrical conductivity, through the equations of Saxton and Rawls^19^ (Table 1).

**Table 1:** Physical and chemical characteristics of the soil and hydrological constants

Parameter	Value
Sand^a^	76.0%
Silt^a^	4.6%
Clay^a^	19.4%
Organic matter^a^	1.9%
Electric conductivity 1:2^a^	0.95 dS m^−1^
Active limestone^a^	5.6%
pH^a^	8.4
Cation‐exchange capacity^a^	14.8 cmol kg^−1^
Kjeldahl N^a^	0.12%
Olsen P_2_O_5_ ^a^	38.9 ppm
Exchangeable K^a^	302 ppm
Wilting point^b^	12.9%
Field capacity^b^	20.0%
Saturation^b^	41.0%
Available water^b^	7.0%

^a^ Measured value.

^b^ Calculated value (Saxton and Rawls)^1^.

The local climate is temperate, characterized by cool, wet winters and hot, dry summers, with the average temperature of the warmest month above 22 °C (Mediterranean climate Csa – according to the Köppen–Geiger classification). Average temperatures in Sicily have shown an upward trend^20^ and this increase is part of a broader pattern of climate change affecting many regions globally.

### Experimental design, fertilization and crop management

**Figure 1:**
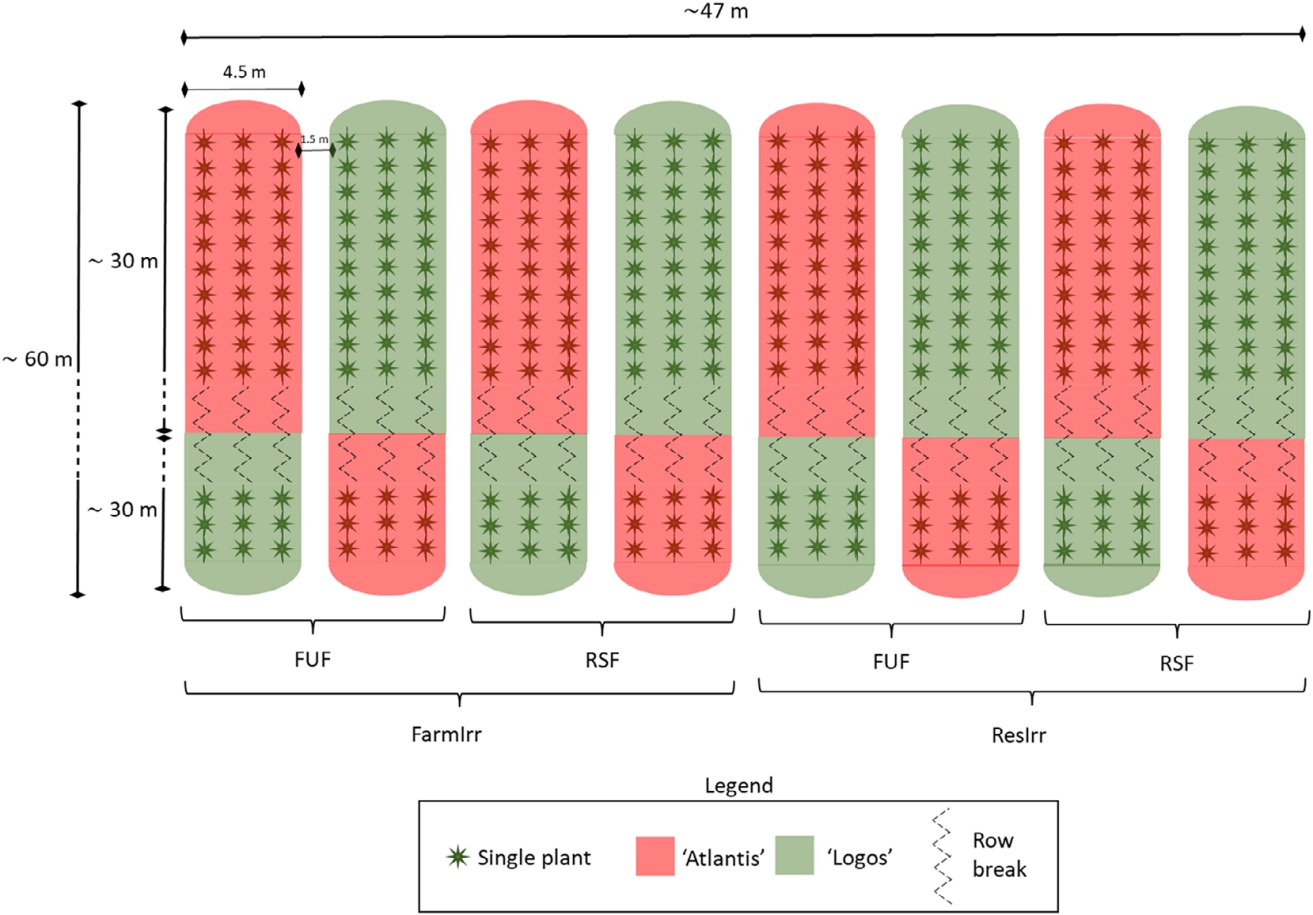
Schematic representation of the experimental field. Four replications were established in each greenhouse tunnel. FUF, farmer's usual fertilization; RSF, researchers' suggested fertilization; FarmIrr, farmer's irrigation; ResIrr, researchers' suggested irrigation.

A split plot design was adopted for testing the effect of two irrigation levels and two fertilization plans on yield, physical traits and mineral content on two commercial zucchini genotypes (2 × 2 × 2, in total eight treatments). Each treatment consisted of four replicates. The year effect was also considered as a main factor. Before transplanting, the soil was ploughed to a depth of 30 cm, then harrowed and finally ridged. On the ridges, a dark plastic mulch (low‐density polyethylene, LDPE) was applied, in order to contain weeds and soil water evaporation.^21^ The field was divided into eight greenhouse tunnels (each 4.5 m wide, 60 m long and 2.5 m high), with an overall experimental area of ~2800 m^2^ (Fig. 1). The distance between plants in the rows was 0.7 m, and that between rows was 1.5 m. Four tunnels on one side were irrigated with higher water volume than on the other side (irrigation management is explained in detail in the next subsection). Four tunnels were provided with the farmer's usual fertilization plan (FUF), the remaining with the researchers' suggested fertilization plan (RSF) (Fig. 1). Soil analyses, carried out just before the experimentation of the first year, showed a high Olsen phosphorus value (38.9 ppm) (Table 1), but its availability was limited by a rather high content of active limestone (5.6%) and alkaline pH (8.4). Exchangeable K amounted to 302 ppm; this value, combined with a cation exchange capacity of 14.8 cmol kg^−1^ (Table 1), indicates abundance and availability of potassium. According to the Sicilian integrated production regulations,^22^ the crop uptake of N, P_2_O_5_ and K_2_O for a zucchini production between 40 and 60 t ha^−1^ amounts to respectively 200, 70 and 350 kg ha^−1^. The FUF was found to be reasonable as regards P_2_O_5_ (100 kg ha^−1^) and K_2_O (200 kg ha^−1^) doses (Table 2), because both of them were consistent with the expected crop uptakes and soil nutrient availability discussed above.

**Table 2:** Fertilization provided according to the farmer's practice and the researchers' plan

Fertilizer	Nutrient	Nutrient doses per each application (kg ha^−1^)						No. fertigations		Nutrient doses applied (kg ha^−1^)		
		Before transplanting		1st phase (fertigation)		2nd phase (fertigation)		1st phase	2nd phase			
		FUF	RSF	FUF	RSF	FUF	RSF			Nutrient	FUF	RSF
Fluid‐hydrolyzed animal epithelium^a^	N	0		12.0	6.7	12.0	6.3	8	20	N	336	180
Soft ground rock phosphate^b^	P_2_O_5_	100		0		0				P_2_O_5_	100	
Potassium sulfate^a^	K_2_O	0		10.2		5.9				K_2_O	200	
Magnesium sulfate^a^	MgO	0		2.1		1.3				MgO	43	
Iron chelate^a^	Fe	0		12.0 × 10^−3^		0				Fe	4.5 × 10^−2^	

1st phase, for 8 weeks starting from transplanting; 2nd phase, for 10 weeks after 1st phase; FUF, farmer's usual fertilization; RSF, researchers' suggested fertilization.

^a^ Provided with fertigation.

^b^ Provided just one time before transplanting.

Conversely, FUF N fertilization, at 336 kg ha^−1^, exceeds the actual requirements of the crop when considering total N and the contribution of soil organic matter (Table 1). The coarse texture of the soil further worsens this issue by promoting the leaching of excess nitrates, which raises environmental concerns. Moreover, the fertigation method applied can enhance nitrogen use efficiency, supporting one of the hypotheses of this paper: that applying less nitrogen can be achieved without compromising crop yields. For these reasons, RSF provided for a sharply lower N fertilization, amounting to 180 kg ha^−1^.

The number of fertilizations, their frequency and the way the fertilizers were supplied according to the two plans are indicated in detail in Table 2. Two zucchini cultivars, namely ‘Atlantis’ and ‘Logos’, were utilized. Fruits were sampled from each of the replicates. The experimentation was carried out on the same plot for two cultivation cycles in two different years, separated by 1 year during which a mixture of grasses, legumes and cruciferous plants was cultivated and subsequently incorporated into the soil as green manure. This discontinuity was necessary because the farm operates organically and crop rotations are mandatory. The transplanting dates were 3 January 2021 in the first year (1st Yr) and 27 October 2022 in the second year (2nd Yr); the cultivation cycle was 117 days in 1st Yr and 120 days in 2nd Yr. In both years, fruit harvesting was conducted in a staggered manner (every 3 days at commercial stage) and lasted approximately 1 month.

### Irrigation management and water productivity

Irrigation was provided with a drip irrigation system, with emitters having a flow rate of 1.14 L h^−1^ and spaced 20 cm apart. Two levels of irrigation were compared: one provided according to the farmer's experience (hereafter referred to as FarmIrr) and the other suggested by researchers (ResIrr), which was the 75% of FarmIrr. The usual farmer's irrigation management has been based solely on observations of the plants, ensuring that they never experienced stress. The farmer has never estimated the water consumption of the crop to adjust the irrigation volumes. Therefore, the rationale of the ResIrr was to provide an easy‐to‐manage alternative to FarmIrr for verifying the researchers’ hypothesis that the farmer provided an irrigation volume exceeding the crop's water requirements. Since the soil is sandy, the farmer has developed an irrigation plan with small irrigations in terms of water volume (4 mm each). In ResIrr, the water volume of each irrigation was 3 mm. In order to evaluate the performance of the two irrigation managements, a soil water balance was calculated on a daily basis considering the water inputs (irrigation and soil moisture) and losses (evapotranspiration). Soil moisture in the top 60 cm before transplanting was determined by the gravimetric method. Daily soil water balances were calculated applying the following constraints:

1. Soil water content (SWC) cannot be higher than field capacity (FC); therefore

if SWC > FC, then SWC = FC

2 SWC cannot be lower than the wilting point (WP), which was taken as the zero of the balance; therefore

if SWC < WP, then SWC = WP

Reference evapotranspiration (ET_0_) was determined using the Hargreaves formula, because it can be filled just with temperature and extraterrestrial radiation and, therefore, it is the most practical for low‐cost plastic greenhouses in Mediterranean climatic regions.^23^ According with Fernández *et al*.,^23^ the Hargreaves formula was corrected by multiplying the extraterrestrial radiation by a transmission coefficient of the tunnel covering material in the photosynthetically active wavelengths; for ethylene‐vinyl acetate this was 0.89.^24^ Since the ridges were mulched with dark plastic film (LDPE), evaporation losses were considered negligible. For this reason, the ET_0_ was multiplied by the basal crop coefficients (K_cb_) provided by FAO paper 56,^25^ in order to obtain the maximum crop evapotranspiration (ET_c_).

The readily available soil water (RAW) for zucchini is considered 50% of the total available water (TAW).^25^ When the RAW was depleted, the water stress coefficient K_s_ was applied to adjust the ETc.^25^ K_s_ was calculated as follows:

$$K_{s}=\frac{\mathrm{TAW}-D_{r}}{\mathrm{TAW}-\mathrm{RAW}}$$

where D_r_ represents depleted water, indicating the water shortage relative to field capacity. At field capacity, D_r_ = 0. The formula above applies when D_r_ > RAW.

Air temperatures utilized for ET_c_ calculation were recorded by an agrometeorological station (Daiki Analytics, Niscemi, Italy). The irrigation water use productivity (IWatP) was calculated and expressed as the dry matter (DM) of fruits obtained with the unit volume of water provided with irrigation (kg DM m^−3^).^26^

### Thermal resources

In order to evaluate the effect of the ‘Year’ factor on the yields, the sum of the growing degree days (GDD) of each cultivation cycle was calculated according to NeSmith,^27^ by applying the following formula:

$$\sum \mathrm{GDD}=\sum \left[\left(T_{\min}+T_{\max}\right)/2\right]-T_{\text{base}}$$

where *T*_min_ is the daily minimum temperature, *T*_max_ is the daily maximum temperature, and *T*_base_ is the minimum cardinal temperature of 8 °C. To that formula, the following conditions were applied:

1. temperatures below minimum cardinal temperature do not affect growth; therefore

if *T*_min_ < 8 °C, then *T*_min_ = 8 °C

2 32 °C is the maximum cardinal temperature; therefore

if *T*_max_ > 32 °C, then *T*_max_ = 32 °C – [2 × (*T*_max_ – 32 °C)]

The second condition means that the maximum daily temperatures exceeding the maximum cardinal temperature were not only cut off but also subtracted from the GDD in order to consider them as a stress factor.^27^

## YIELDS AND PHYSICAL AND COLOR TRAITS OF FRUITS

Fruit sampling was carried out every 7–10 days from each treatment and replicate (*n* = 384 fruits). In both 1st and 2nd Yr, the yields of each treatment were estimated by multiplying the number of harvested fruits in each period by the fruit average weight of each period. Fresh weight, dry weight (in oven at 105 °C), color and firmness of each fruit were recorded. The latter was evaluated with a digital fruit firmness tester (TR Turoni, Forli, Italy). Colorimeter readings of L*, a* and b* values were carried out with a CR‐300 Chroma Meter (CR‐300, Konica Minolta, Tokyo, Japan) on four points of each zucchini fruit. The different color indexes were calculated according to the following equations:

$$\mathrm{Hue}=\tan^{-1}{\left(b^{*}/a^{*}\right)}^{2}$$

$$\text{Chroma}={\left(a^{*2}+b^{*2}\right)}^{0.5}$$

### Fruits mineral content and nitrogen use efficiency

Nine minerals (P, K, Mg, Ca, Na, Fe, Mn, Zn and Cu) were also analyzed, as reported by Lombardo *et al*.^28^ An amount (~1 g ± 0.1) of oven‐dried material, per replicate and treatment, was placed in a muffle furnace at 550 ± 2 °C for 24 h. After cooling at room temperature in a desiccator, P was estimated according to the molybdovanadate colorimetric method 986.24^29^ using a Shimadzu 1601 UV–visible spectrometer (Shimadzu Corp., Kyoto, Japan). The other minerals were analyzed using a PerkinElmer (Norwalk, CT, USA) AAnalyst 200 atomic absorption spectrometer equipped with a multi‐element hollow cathode lamp; a deuterium background correction system was used, after ashing about 1 ± 0.1 g of oven‐dried material. Each individual mineral in the sample was quantified from its calibration curve and data were expressed as g or mg kg^−1^ DM. All analyses were performed in triplicate. All the reagents and solvents were purchased from Sigma‐Aldrich (Milan, Italy) and were of analytical grade. Bidistilled water was used throughout this research. Total nitrogen in the fruits was determined by the Kjeldahl method.^30^ Nitrogen content of fruits was used to assess the nitrogen use efficiency (NUE) as the fraction of fertilizer N that was utilized and allocated to yield N,^31^ with the following formula:

$${\mathrm{NUE}}_{\text{crop}}=\frac{\text{Fruits}\;N}{\text{Fertilizer}\;N}$$

### Statistical analysis

The dependent variables (yields, IWatP, NUE_crop_, fruit mineral content, and physical and color traits of fruits) were subjected to analysis of variance (ANOVA),^32^ and the means for each trait were separated by Fisher's least significance difference test, applying a threshold of 0.05. Homogeneity of variance and normality were respectively verified with the Bartlett and Shapiro–Wilk tests. A preliminary four‐way ANOVA was performed, taking into account the independent variables, namely two irrigation levels (*I*), two fertilization levels (*F*), two genotypes (*G*), and 2 years (*Y*) (*I* × *F* × *G* × *Y*). Subsequently, only the significant interactions were considered; in the absence of significant interactions, the main effects were examined. For IWatP and minerals, four‐way ANOVAs were conducted. Two three‐way ANOVAs were performed for both yields and NUE_crop_. Only for the physical and color traits of fruits, where there were no interactions among factors, one‐way ANOVAs were conducted for the main factors. The statistical software CoStat Version 6.451 (CoHort Software, Birmingham, UK) was used.

## RESULTS AND DISCUSSION

### Soil water content and air temperature inside the tunnels

Before transplanting, the soil moisture in the top 60 cm profile in both 1st and 2nd Yr was in the RAW range, due to the previous precipitations. Despite higher average temperatures being recorded in 2nd Yr than in 1st Yr, seasonal irrigation volumes provided in the 1st Yr were higher than those in the 2nd Yr (Table 3). This is because in the 1st Yr the period of greatest evapotranspiration demand for plants occurred in spring (March–April), while in the 2nd Yr it occurred in winter (January–February). According to the water balances, in both 1st and 2nd Yr, a portion of the FarmIrr was lost through deep percolation (on average, 56 mm) (Table 3). The ResIrr did not result in losses in the 1st Yr, during which, on the other hand, plants experienced moderate water stress on some days (Table 3).

**Table 3:** Average air temperatures under greenhouse tunnels, components of soil water balance, and water stress days in the years of experimentation

Year	Average temperature (°C)			GDDs (°C)	SIV (mm)		ET_c_ (mm)		DP (mm)		Days with SWC < RAW	
	*T*_max_	*T*_mid_	*T*_min_		FarmIrr	ResIrr	FarmIrr	ResIrr	FarmIrr	ResIrr	FarmIrr	ResIrr
1st Yr	21.7	15.1	9.3	913	240	180	208	203	45	0	0	6
2nd Yr	22.5	17.6	11.1	1089	200	150	124	124	67	18	0	0

DP, deep percolation; ET_c_, crop evapotranspiration; GDDs, growing degree days; RAW, readily available water; SIV, seasonal irrigation volume; SWC, soil water content.

### Fruit yield

**Figure 2:**
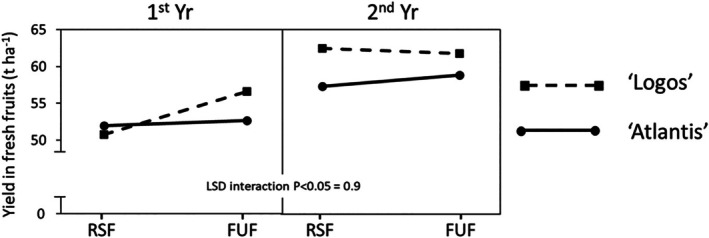
Effect of three‐way ‘Fertilization × Genotype × Year’ interaction on fresh fruit yields. 1st Yr = first year; 2nd Yr = second year. FUF, farmer's usual fertilization; RSF, researchers' suggested fertilization.

A three‐way ‘Fertilization × Genotype × Year’ interaction (Fig. 2) was observed. In 1st Yr, only ‘Logos’ responded to the higher level of N fertilization, with yield increasing from 50.8 t ha^−1^ with RSF to 56.6 t ha^−1^ with FUF (Fig. 2). In contrast, in 2nd Yr, only ‘Atlantis’ demonstrated a response to the elevated fertilization levels, increasing its yield from 57.3 to 58.9 t ha^−1^ (Fig. 2). Changing perspective, the farmer can halve nitrogen fertilizer for ‘Atlantis’ without compromising yields in late cycles, such as in the 1st Yr, characterized by higher temperatures. With ‘Logos’, the farmer can similarly reduce nitrogen fertilization in earlier cycles, such as in the 2nd Yr, with lower average temperatures. Both genotypes yielded more in 2nd Yr, probably because the latter provided the plants with higher thermal resources than 1st Yr (1089 *vs*. 913) (Table 3), in which temperatures above the cardinal maximum were more frequent. Upon closer examination, the 2nd Yr significantly enhanced the productivity of ‘Logos’ compared to ‘Atlantis’. Specifically, ‘Logos’ exhibited a yield increase of 15.6% from 1st Yr to 2nd Yr, whereas ‘Atlantis’ demonstrated a yield increase of 11.0% (Fig. 2). These differences should not surprise, since weather variations explain over 50% of crop yield variability.^33^ Generally, vegetables experience temperature‐dependent developmental stages, and high temperatures accelerate these processes, potentially shortening crop duration but risking lower yields.^34^

**Figure 3:**
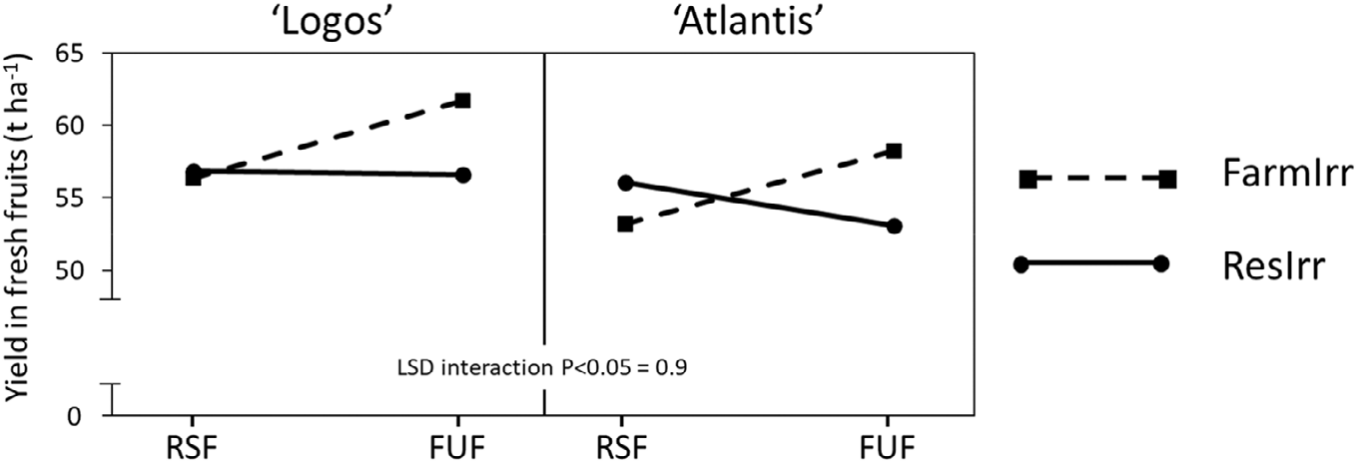
Effect of three‐way ‘Irrigation × Fertilization × Genotype’ interaction on fresh fruit yields. FUF, farmer's usual fertilization; FarmIrr, farmer's irrigation; ResIrr, researchers' suggested irrigation; RSF, researchers' suggested fertilization.

A three‐way ‘Irrigation × Fertilization × Genotype’ interaction was also observed (Fig. 3). Under ResIrr, no different yields corresponded to different fertilization plans in ‘Logos’ (~56.7 t ha^−1^), while with FarmIrr the FUF nitrogen dose enhanced the production (+9.5%) (Fig. 3). Conversely, a crossover interaction between irrigation and fertilization was observed for ‘Atlantis’; in fact, under FarmIrr, FUF enhanced the yield (from 53.2 to 58.2 t ha^−1^), while reducing it under ResIrr (−5.3%) (Fig. 3). In both genotypes, the highest and lowest yields were obtained with the highest N dose. This may seem contradictory, but it must be considered that (1) excess nitrogen in the soil can depress production performance, and (2) nitrogen can be leached through water percolation. In other words, excess irrigation may have leached out some of the excess nitrogen, bringing its content close to the optimum. After all, excessive application of nitrogen fertilizers in greenhouse vegetable production leads to significantly higher nitrogen losses than production gains,^35^ a situation worsened by frequent over‐irrigation events.^36^

On average, the yields obtained in this study were higher than those reported by Toscano *et al*.^37^ under organic farming conditions.

### Irrigation water productivity

**Figure 4:**
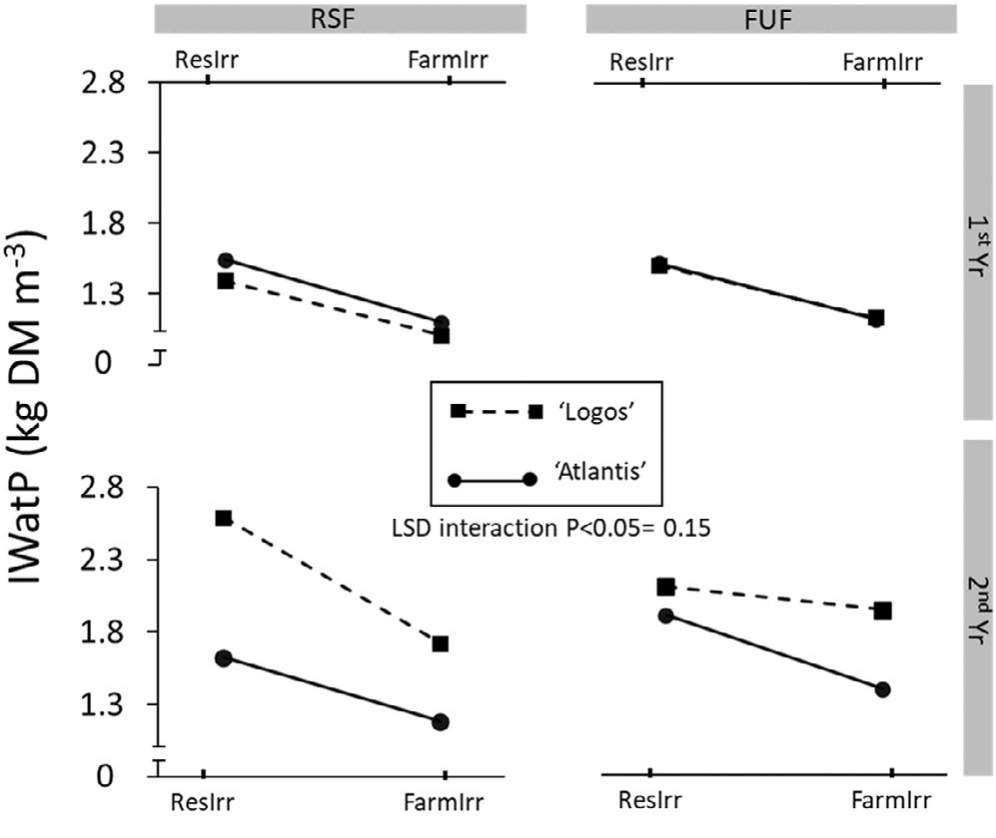
Effect of four‐way ‘Irrigation × Fertilization × Genotype × Year’ interaction on irrigation water productivity (IWatP). 1st Yr, first year; 2nd Yr, second year; FarmIrr, farmer's irrigation; FUF, farmer's usual fertilization; ResIrr, researchers' suggested irrigation; RSF, researchers' suggested fertilization.

A significant four‐way ‘Irrigation × Fertilization × Genotype × Year’ interaction was observed. Since higher yields were obtained in 2nd Yr with lower irrigation volumes, it is not surprising that IWatP was higher in the 2nd Yr (1.81 kg DM m^−3^) than in the 1st Yr (1.29 kg DM m^−3^) (Fig. 4). In both 1st Yr and 2nd Yr the IWatP of ResIrr was higher than that of FarmIrr (on average + 34%) (Fig. 4), while fertilization did not have a significant effect (Fig. 3). It is interesting to note that in 1st Yr there was no significant difference between IWatPs achieved with the two genotypes, while in 2nd Yr, ‘Logos’, with an average of 2.1 kg DM m^−3^ across the treatments, promoted a greater production (+36%) of fruits DM per unit of water supplied with irrigation (Fig. 4). In this case there may have been a synergistic effect between the genotype and the higher accumulation of GDDs.

Rouphael and Colla^3^ reported values for the ratio between fresh fruit mass and transpired water in greenhouse‐grown zucchini ranging from 31.5 to 50.5 kg m^−3^. Similarly, Contreras *et al*.^38^ found values between 42.6 and 47.3 kg m^−3^. These results are perfectly compatible with the IWatPs of the present study, which, reported as fresh weight, range from 22.1 to 40.1 kg m^−3^. The latter also fall within the broad range of values (from 4.3 to 57.5 kg m^−3^, from the wettest to the driest treatment) presented by Zotarelli *et al*.,^39^ who related fresh fruit yields to irrigation water. The IWatPs found in the present study are four times higher than those reported by Darouich *et al*.^40^ in a Mediterranean environment, using the same irrigation method (drip irrigation), but under open‐air conditions and without mulching.

### Nitrogen use efficiency

**Figure 5:**
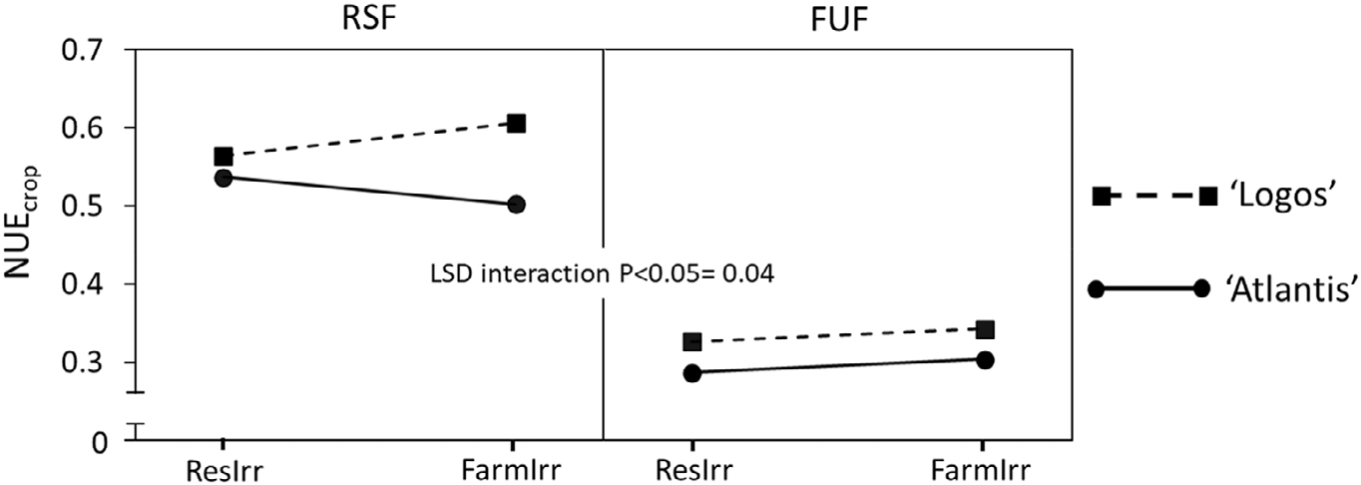
Effect of three‐way ‘Irrigation × Fertilization × Genotype’ interaction on nitrogen use efficiency (NUE_crop_). 1st Yr, first year; 2nd Yr, second year; FarmIrr, farmer's irrigation; FUF, farmer's usual fertilization; ResIrr, researchers' suggested irrigation; RSF, researchers' suggested fertilization.

**Figure 6:**
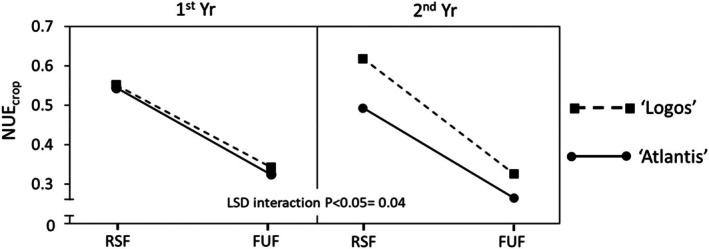
Effect of three‐way ‘Fertilization × Genotype × Year’ interaction on nitrogen use efficiency (NUE_crop_). 1st Yr, first year; 2nd Yr, second year; FarmIrr, farmer's irrigation; FUF, farmer's usual fertilization; ResIrr, researchers' suggested irrigation; RSF, researchers' suggested fertilization.

A significant three‐way ‘Irrigation × Fertilization × Genotype’ interaction was found (Fig. 5). As expected, with the application of RSF, significantly higher NUE_crop_ values were recorded than with FUF (on average, 0.55 *vs*. 0.31) (Figs 5 and 6). With RSF, the NUE_crop_ of ‘Logos’ increased with the rise in irrigation (from 0.56 to 0.61), whereas that of ‘Atlantis’ decreased (from 0.54 to 0.50) (Fig. 5). The RSF not only reduces the environmental footprint associated with excessive nitrogen application but also promotes sustainability by increasing the proportion of nitrogen that is effectively utilized in fruit production.

A three‐way ‘Fertilization × Genotype × Year’ interaction was also observed (Fig. 6). While in the 1st Yr the differences between the two genotypes were not statistically significant, in the 2nd Yr they were more pronounced, with ‘Logos’ exhibiting average NUE_crop_ values that were 0.09 higher than those of ‘Atlantis’ (Fig. 6).

NUE_crop_, can vary greatly, depending on the level of irrigation, environmental conditions and, above all, fertilization, from 0.17 to 0.82.^39^ Contreras *et al*.^38^ expressed NUE as the ratio between yield of fresh fruits and N consumption, and found that from 253 to 282 kg of fresh fruits were obtained per kilogram of N consumed. These results fall in the range observed in the present study, where 168 and 314 kg of fresh fruits per kilogram of N provided were obtained, with FUF and RSF respectively.

### Fruits physical and color traits

According to the ANOVA, significant effects of the main factors were observed on the color and physical traits of the fruits, but no interactions were found. Regardless of the factor, in 2nd Yr average fruit weight was significantly higher than in 1st Yr (247 *vs*. 190 g respectively, on average +30%) (Table 4). This is because, in the 2nd Yr, the market required larger fruits, and the farmer allowed them to grow for a longer time. In this regard, it is important to note that the present work presents the results of an OFE, conducted within the framework of a real farm under real conditions. Regarding the genotypes, ‘Atlantis’ showed a higher dry weight than ‘Logos’ in 1st Yr (5.2 *vs*. 4.8%), but 2nd Yr promoted a higher accumulation of DM in fruits of ‘Logos’ (5.7% *vs*. 4.6%). This could be reflected by the fruit firmness, which, specifically in the 2nd Yr, was significantly higher in ‘Logos’ fruits. The latter were brighter (*L* value) and greener (hue value) in the 1st Yr than in the 2nd Yr (Table 4). ‘Atlantis’ fruits showed more intense chroma than ‘Logos’ in both years of experimentation. Fertilization had a statistically significant effect on the fruit weight, but only in the 1st Yr (Table 4). The ResIrr resulted in a higher accumulation of dry matter in the fruits, but only in the 1st Yr. Regardless of the factor, firmness was higher in the 2nd Yr (on average, 0.13 *vs*. 0.10 kg cm^−2^) (Table 4), probably due to the higher ripeness degree of fruits harvested in the 2nd Yr. Saturation (chroma) always showed higher values in the 1st Yr, regardless of the factor (on average, 22.0 *vs*. 18.9). The same trend was observed for hue (on average 135 *vs*. 132.7); however, in this case, the differences were not always statistically significant (Table 4). Overall, it can be concluded that the fruits of the two genotypes showed similar peel colors and that the ‘Year’ factor affected color attributes more than the genotypes (Table 4). These results are not unexpected, given that color variability in numerous vegetables has been documented in relation to temperature.^34^

**Table 4:** Fresh weight, dry weight, firmness and color traits of fruits as affected by genotype, fertilization and irrigation in the 2 years (Yr)

Source of variation	Fresh weight (g)		Dry weight (%)		Firmness (kg cm^−2^)		*L*		Hue		Chroma	
	1st Yr	2nd Yr	1st Yr	2nd Yr	1st Yr	2nd Yr	1st Yr	2nd Yr	1st Yr	2nd Yr	1st Yr	2nd Yr
Genotype											
‘Atlantis’	193b	255a	5.2aA	4.6bB	0.10b	0.12aB	35.2	35.5	134.2	132.8	22.5aA	19.4bA
‘Logos’	186b	238a	4.8bB	5.7aA	0.09b	0.14aA	35.0a	32.8b	135.8a	132.6b	21.5aB	18.3bB
Fertilization											
RSF	183bB	253a	5.0	5.0	0.10b	0.13a	35.2a	33.2b	136.4a	132.8b	22.3a	19.1b
FUF	195bA	241a	5.0	5.2	0.09b	0.14a	35.0	35.1	133.6a	132.5b	21.6a	18.6b
Irrigation											
ResIrr	188b	250a	5.2A	5.1	0.10b	0.13a	35.4a	33.2b	134.1	132.6	22.1a	19.1b
FarmIrr	191b	244a	4.9B	5.1	0.10b	0.13a	34.8	35.2	135.9a	132.8b	21.8a	18.6b

In each row, within each trait, different lower‐case letters indicate significantly different values in Fisher's LSD test (*P* < 0.05) between the 1st and 2nd Yr, while different upper‐case letters indicate significant differences within factor in the same year.

FarmIrr, farmer's irrigation; FUF, farmer's usual fertilization; ResIrr, researchers' suggested irrigation; RSF, researchers' suggested fertilization.

### Fruit mineral content

Fruit nitrogen content in 1st Yr was significantly higher than in 2nd Yr (on average 39.7 *vs*. 32.7 g kg^−1^ DM) (Table 5). This fact can be well explained considering that (1) in 2nd Yr the fruits were significantly larger, and that (2) fruit growth occurs not through cell division but primarily through cell growth,^41^ due to vacuole enlargement.^42^ Consequently, at the same dry mass, the content of nitrogenous compounds (e.g., amino acids and nitrogenous bases) was higher in fruits of 1st Yr, which were harvested earlier.^43^ With RSF, the N content in ‘Logos’ fruits increased as the irrigation volume increased in both 1st Yr and especially 2nd Yr (+25%), while that of ‘Atlantis’ decreased, but not significantly (Table 5). However, with RSF, the ranking of the two genotypes was reversed in the two years; this reveals the existence of a complex interaction ‘Genotype × Irrigation × Year’. With FUF, in the first year, the differences in N content between genotypes were not statistically significant (from 39.6 g kg^−1^ of ‘Atlantis’ with ResIrr to 44.1 g kg^−1^ of ‘Logos’ with FarmIrr). In the second year, due to the effect of FUF, the fruits of ‘Atlantis’ increased the nitrogen content with increasing irrigation (+14%), but the exact opposite occurred with ‘Logos’ (−20%). The latter, in fact, yielded more and the nitrogen compounds were distributed in a greater mass of fruits. Nitrogen values found in this study are in line with those found by Rouphael *et al*.^44^

**Table 5:** Macroelements in fruits as affected by year, genotype, fertilization level and irrigation volume.

Macroelement	Y↓	G↓	F→	RSF		FUF	
			I→	ResIrr	FarmIrr	ResIrr	FarmIrr
N (g kg^−1^ DM)	1st Yr	‘Atlantis’		36.45B–D	35.85CD	39.55A–D	40.44A–C
		‘Logos’		38.68A–D	42.04AB	40.19A–C	44.12A
	2nd Yr	‘Atlantis’		37.64B–D	36.37B–D	29.53E–G	33.73D–F
		‘Logos’		27.29G	34.01D‐F	34.98C–E	28.14FG
P (g kg^−1^ DM)	1st Yr	‘Atlantis’		2.12BC	1.97BC	2.25BC	3.49AB
		‘Logos’		1.95BC	2.67B	2.72B	4.11A
	2nd Yr	‘Atlantis’		1.91BC	2.16BC	2.43BC	2.48B
		‘Logos’		2.32BC	1.50C	2.43BC	1.46C
K (g kg^−1^ DM)	1st Yr	‘Atlantis’		38.01AB	33.24A–C	32.43A–C	30.66A–C
		‘Logos’		41.63A	36.82AB	26.98BC	36.36AB
	2nd Yr	‘Atlantis’		22.93C	33.01A–C	32.35A–C	38.00AB
		‘Logos’		20.98C	28.81BC	30.25A–C	26.92BC
Ca (g kg^−1^ DM)	1st Yr	‘Atlantis’		2.34A–C	1.58C	2.85A–C	3.07A–C
		‘Logos’		2.35A–C	1.98BC	3.36A–C	3.39A–C
	2nd Yr	‘Atlantis’		3.65A–C	4.34A	4.05AB	4.20A
		‘Logos’		3.55A–C	3.96AB	3.69AB	4.05AB
Mg (g kg^−1^ DM)	1st Yr	‘Atlantis’		2.44A–D	2.27A–E	2.88AB	2.08A–G
		‘Logos’		2.65A–C	1.70C–G	1.47D–G	2.25A–F
	2nd Yr	‘Atlantis’		1.14G	2.37A–E	2.20A–F	3.07A
		‘Logos’		1.34E–G	1.71C–G	1.88B–G	1.21FG
Na (g kg^−1^ DM)	1st Yr	‘Atlantis’		0.28A–D	0.26B–D	0.27A–D	0.26B–D
		‘Logos’		0.26B–D	0.27A–D	0.26CD	0.25D
	2nd Yr	‘Atlantis’		0.29AB	0.28A–D	0.30A	0.29A–C
		‘Logos’		0.29A–C	0.29A–C	0.29A–C	0.29A–C

Within each macroelement, values that do not share a letter are significantly different in Fisher's LSD test (*P* < 0.05).

1st Yr, first year; 2nd Yr, second year; F, fertilization; FarmIrr, farmer's irrigation; FUF, farmer's usual fertilization; G, genotype; I, irrigation; ResIrr, researchers' suggested irrigation; RSF, researchers' suggested fertilization; Y, year.

Regarding the other macroelements, all responded to the ‘Year’ factor (Table 5). Phosphorus, K and Mg, namely those affecting physiological and biochemical processes in the nucleus and cytoplasm,^45^, ^46^, ^47^ were on average more abundant in 1st Yr fruits, which, as stated before, were smaller than those of 2nd Yr (Table 5). Conversely, in 2nd Yr fruits Ca and Na contents were higher than in 1st Yr, probably because (1) Ca is primarily found in the cell walls, whereas Na is also present in the cell membranes, and (2) cell walls and cell membranes tend to be more developed in larger cells compared to smaller ones.^48^ Additionally, Ca and Na are located in vacuoles,^42^, ^49^ which are larger in larger fruits. Phosphorus was at the center of complex interactions between factors, and its accumulation in fruits was positively influenced by high inputs (FarmIrr and FUF) (Table 5). The average values found in this study of P (2.4 g kg^−1^ DM), K (31.8 g kg^−1^ DM), Ca (3.3 g kg^−1^ DM) and Mg (2.0 g kg^−1^ DM) are in line with those of Martínez‐Valdivieso *et al*.^50^ In other studies,^44^, ^51^ the P, K and Mg contents were higher, but fruits were smaller (on average between 120 and 150 g) and, in any case, yields were lower. The microelement content of the fruits was generally affected by the ‘Year’ effect (Table 6). In any case, all the microelements were at the center of more or less complex interactions between factors (Table 6). The mean values recorded for Fe (29.1 mg kg^−1^ DM), Mn (17.4 mg kg^−1^ DM), Zn (11.0 mg kg^−1^ DM) and Cu (1.9 mg kg^−1^ DM) were very close to those found by Martínez‐Valdivieso *et al*.,^50^ but lower than those found by Rouphael *et al*.^44^ for the same reasons explained above concerning macroelements. The data presented above further demonstrate that changing the conditions in the greenhouse affects the physiological processes that lead to differences in the composition of vegetable products.^34^

**Table 6:** Microelements in fruits and their ash content as affected by year, genotype, fertilization level and irrigation volume

Microelement/ash			F→	RSF		FUF	
	Y↓	G↓	I→	ResIrr	FarmIrr	ResIrr	FarmIrr
Fe (mg kg^−1^ DM)	1st Yr	‘Atlantis’		29.70AB	29.45A–C	30.68AB	30.69AB
		‘Logos’		31.14AB	30.64AB	30.40AB	28.11A–C
	2nd Yr	‘Atlantis’		32.46A	30.11AB	25.39BC	29.48AB
		‘Logos’		26.65A–C	28.98A–C	29.48AB	22.58C
Mn (mg kg^−1^ DM)	1st Yr	‘Atlantis’		14.86D	14.92D	15.06CD	17.88AB
		‘Logos’		14.83D	14.93D	14.31D	17.38BC
	2nd Yr	‘Atlantis’		18.19AB	19.31AB	18.89AB	20.27A
		‘Logos’		18.47AB	19.31AB	19.44AB	19.99A
Zn (mg kg^−1^ DM)	1st Yr	‘Atlantis’		11.62A	10.16A	7.67B	11.72A
		‘Logos’		7.97B	11.03A	10.50A	13.44A
	2nd Yr	‘Atlantis’		12.72A	12.67A	6.26B	13.72A
		‘Logos’		7.84B	12.30A	13.35A	12.23A
Cu (mg kg^−1^ DM)	1st Yr	‘Atlantis’		1.59B	1.81AB	1.64B	1.70B
		‘Logos’		1.50B	1.52B	1.48B	1.79B
	2nd Yr	‘Atlantis’		2.00AB	2.00AB	2.23AB	2.23AB
		‘Logos’		3.03A	2.11AB	2.46AB	1.31B
Ash (g kg^−1^ DM)	1st Yr	‘Atlantis’		64.94A–C	61.18A–C	53.60BC	60.62A–C
		‘Logos’		67.33A–C	73.04AB	58.82BC	69.58A–C
	2nd Yr	‘Atlantis’		75.13AB	75.14AB	67.40A–C	65.88A–C
		‘Logos’		43.63C	79.84AB	86.47A	73.58AB

Within each variable (microelement or ash), values that do not share a letter are significantly different in Fisher's LSD test (*P* < 0.05).

1st Yr, first year; 2nd Yr, second year; F, fertilization; FarmIrr, farmer's irrigation; FUF, farmer's usual fertilization; G, genotype; I, irrigation; ResIrr, researchers' suggested irrigation; RSF, researchers' suggested fertilization; Y, year.

### Study limitations

Despite the valuable findings of this study, two limitations should be acknowledged. First is the relatively short duration of the experiment, which lasted 2 years. However, these 2 years were separated by a 1‐year gap, as indicated in the ‘Materials and Methods’ section, above. This break was necessary due to the organic farming system, which required the zucchini cultivation to be integrated into a crop rotation. Therefore, while the experimental period spanned 2 years, the overall time frame was 3 years. The second limitation pertains to the differing transplant times in the 2 years of the experiment. This variation influenced the environmental conditions, particularly temperature. It is important to note, however, that the study was conducted within the context of a real farm (OFE), which operated under specific needs and constraints, such as market demands and labor availability.

### Future research directions

In the context of ongoing climate change, species are shifting to higher latitudes.^52^ Therefore, crops traditionally cultivated in Mediterranean environments, such as zucchini, should undergo multi‐site trials under varying conditions to address the unpredictability of climate change. Research should explore alternative nutrient sources or amendments, such as biochar, which has been shown to enhance soil organic matter, fruit yield, nitrogen use efficiency, and overall agronomic efficiency in zucchini plants grown in calcareous sandy soils, such as that in the present study.^53^ Moreover, utilizing internet‐based technologies for real‐time monitoring and control of agricultural parameters can optimize inputs, enhancing vegetable production efficiency.^54^ In organic agriculture, alternatives to synthetic agrochemicals are essential, prompting research into the genetic composition of plants to improve resistance to pathogens. The decreasing cost of sequencing has accelerated crop genomic data accumulation, offering significant opportunities for breeders. The zucchini genome sequence serves as a crucial resource for identifying traits relevant to agronomy, facilitating assessments of genetic variations during breeding and enhancing resistance to pathogenic infections, which are a major issue in organic farming.^55^ All these sustainable solutions can be tested and disseminated in the frame of OFEs, as they can solve farmers' recognized issues.

## CONCLUSIONS

The present study offers insights into enhancing the sustainability of zucchini cultivation in Mediterranean regions, showcasing the effectiveness of OFE as a key method for generating applicable results for farmers and reliable findings for researchers. The findings corroborate the hypothesis presented in this paper, indicating that farmers can achieve substantial yields, while significantly reducing inputs, specifically by 46% for nitrogen fertilizer and 25% for irrigation water, compared to their typical usage practices. The mitigation of environmental costs and the improvement of economic benefits are further confirmed by the higher NUE_crop_ and IWatP values obtained with reduced‐input practices. The study reveals that ‘Logos’ outperformed ‘Atlantis’ utilizing both fertilization and thermal resources more effectively. This confirms that the choice of genotype impacts sustainability. Furthermore, selecting the optimal planting time is crucial for enhancing yields and ensuring better crop performance, particularly in the context of climate change. The importance of the ‘Year’ factor is underscored by its influence on all qualitative characteristics of the fruits, even surpassing that of the ‘Genotype’ factor. A farmer‐centered framework for the dissemination of sustainable agricultural practices provides an effective approach for introducing innovations to farmers and their engagement through OFEs. This participatory approach not only fosters collaboration between researchers and farmers but also ensures that innovations are directly aligned with the needs and challenges faced in the field. Ultimately, technologies that address the real challenges faced by farmers are more likely to be readily adopted.

## Data Availability

The data that support the findings of this study are available on request from the corresponding author. The data are not publicly available due to privacy or ethical restrictions.
